# Facebook use and its predictive factors among students: Evidence from a lower- and middle-income country, Bangladesh

**DOI:** 10.3389/fpsyt.2022.945802

**Published:** 2022-07-29

**Authors:** Firoj Al-Mamun, Ismail Hosen, Mark D. Griffiths, Mohammed A. Mamun

**Affiliations:** ^1^CHINTA Research Bangladesh, Savar, Bangladesh; ^2^Department of Public Health and Informatics, Jahangirnagar University, Savar, Bangladesh; ^3^Department of Public Health, University of South Asia, Dhaka, Bangladesh; ^4^Psychology Department, Nottingham Trent University, Nottingham, United Kingdom

**Keywords:** COVID-19, problematic Facebook use, Facebook addiction, online behavior, students

## Abstract

**Background:**

Facebook is a popular social networking site in the modern world. It has an adverse effect such as impairing daily health and psychological health and also interpersonal relationships when the use becomes problematic.

**Aims:**

To examine problematic Facebook use (PFU) and its predictors among Bangladeshi students during the COVID-19 pandemic.

**Method:**

A cross-sectional online survey was conducted among 601 Bangladeshi students and collected data related to socio-demographic information, behavioral health, internet use behavior, depression, anxiety and problematic Facebook use [assessed using the Bergen Facebook Addiction Scale (BFAS)]. The data were analyzed using descriptive (frequencies and percentages) and inferential statistics (independent sample *t*-tests, one-way ANOVAs, correlations, and multivariable linear regression).

**Results:**

The results indicated that 29.1% of participants were problematic Facebook users (using cutoff ≥18 out of 30). Medical college students had higher mean score on PFU than other students (*p* < 0.001). In addition, the mean score of PFU was significantly higher among the students who were in a relationship (*p* = 0.001), did not engage in physical activity (*p* < 0.001), used the internet more than 5 h per day (*p* < 0.001), used social media (*p* < 0.001), and had depression or anxiety symptoms (*p* < 0.001). PFU was significantly associated with depression and anxiety among the whole sample. Predictive factors for PFU included relationship status, daily internet use time, gaming, social media use, depression, and anxiety. The model predicted almost 33.2% variance for PFU.

**Conclusions:**

Findings suggest interventions should be implemented for students with a special focus on medical students who had higher score of PFU than other types of students.

## Introduction

Millions of individuals worldwide have been affected by the disruptive consequences of the COVID-19 pandemic. To combat the pandemic, governments implemented preventive actions to minimize the spread of the virus such as national and local lockdowns, closing all educational institutions and introducing online teaching, shutting down non-essential businesses, and enforcing spatial distancing. Such measures were also introduced in Bangladesh (where the present study was carried out) (1, 2). Such measures have led to a situation of increased social media use to stay connected with work colleagues, to engage in education activities, and to interact socially with friends and acquaintances (3, 4).

Facebook is widely considered as one of the most popular social networking sites globally, and it has had a significant impact on interpersonal communication (5). However, problematic Facebook use (PFU) occurs when the engagement becomes uncontrollably excessive and has a negative impact and clinically impairs daily activities, interpersonal relationships, and psychological well-being (6, 7). Moreover, a recent study reported that students who were problematic Facebook users scored significantly higher than non-problematic Facebook users on the meaninglessness of effortfulness, belief in fate, and belief in an unjust world (8). During the COVID-19 pandemic, problematic social media use appears to have increased because individuals' use of technologies has increased as a result of home confinement and/or staying at home for quarantining purposes (9, 10) and technology-associated risky behaviors have been reported (11).

Dhaka, the capital of Bangladesh, had the second highest Facebook use among all cities globally in 2018 (12) and suggests PFU might be a growing issue (13). In March, 2020, it was reported that there were more than 37 million Facebook users in Bangladesh, which increased to more than 44 million by the end of the year (14). One-quarter of the Bangladeshi population are currently active users of Facebook, and young adults (aged 18–24 years) are the largest group of users (14). The number of adult users using social networking sites has increased during the COVID-19 pandemic because of switching to online learning and fewer opportunities to meet socially (10). For instance, an online survey among Italian adults during the COVID-19 pandemic lockdown reported social networking use had increased significantly (10), as has been found in other studies [e.g., (15, 16)].

Excessive Facebook use has the potential to interrupt learning processes and can result in a negative impact on academic performance (17). Moreover, problematic Facebook use can be deleterious to physical and psychological wellbeing among a minority of individuals. For instance, PFU has been associated with depression, anxiety, stress, low self-esteem, personality disorder, and (in extreme cases) suicidality (18–20). One study conducted during the pandemic in Bangladesh related to social media use (as opposed to PFU) by Hossain et al. (9), reported that increased social media exposure over 4 h per day led individuals to be more anxious than individuals who used it for <2 h daily.

Despite increasing concern, there have been few studies assessing PFU in Bangladesh during COVID-19 although two studies were conducted prior to the pandemic. Mamun and Griffiths (13) surveyed 300 Bangladeshi university students and reported a PFU prevalence rate of 39.7%. This study reported that PFU was associated with being single, having less involvement in physical activities, sleep disturbance (when individuals sleep more or <6–7 h of sleep nightly), time spent on Facebook (≥5 h per day), and depression symptoms. Another study by Sayeed et al. (21) surveyed 404 university students and reported a similar PFU prevalence rate of 36.9%. In this study, PFU was associated with having relationship break-ups, having history of domestic violence, having stressful life events, suffering from sleep disturbance (i.e., more than 8 h sleep status compared to 6–8 h normal status), spending more than 5 h daily time on Facebook, and having symptoms of depression (21).

Internet use appears to have increased during the pandemic because the time spent indoors has greatly increased due to self-isolation, lockdowns and quarantines. Moreover, there appears to be far more research on Facebook addiction in high-income countries than in lower- and middle-income (LMIC) countries probably because internet use is more widespread than high-income countries. Therefore, the present study was carried out in a LMIC country. Additionally, PFU has not been examined in Bangladesh during the pandemic. Given the lack of research, the present study investigated PFU among Bangladeshi students to (i) examine the prevalence of problematic Facebook use during the COVID-19 pandemic, and (ii) identify the correlates and predictive factors of PFU. As the study was exploratory, there were no specific hypotheses.

## Methods

### Study procedure and participants

A web-based survey was carried out among the Bangladeshi students between October 7 and November 2, 2020 through the use of *Google Forms*. The Checklist for Reporting Results of Internet E-Surveys was utilized in conducting the survey. A structured questionnaire was used for this study recruiting participants from social media such as Facebook, WhatsApp, etc. in Bangladesh where students interact with each other. A convenience sampling technique was utilized to collect data and the study inclusion criteria were being a Bangladeshi student, studying at high school, college or university, having access to the internet, and wanting to voluntarily participate in the study. A total of 617 individuals completed the survey but after removing the incomplete responses, 601 survey responses were considered for final analysis. The survey included questions concerning socio-demographics, behavioral health, and internet use behaviors, along with psychometric scales assessing depression, anxiety, and problematic Facebook use.

### Ethical considerations

Informed consent was outlined in the description of the study purpose. No incentives (e.g., monetary rewards, prizes, or non-monetary incentives) were offered to participate in the survey. The respondents were only able to participate in the survey after they agreed to the online consent (that adhered to the guidelines of the Helsinki Declaration, 1975). Participants were assured about the anonymity and confidentiality of the data and they had the full right to withdraw their responses at any time during the survey time. Formal ethics permission was also provided by the ethical review committee of Institute of Allergy and Clinical Immunology of Bangladesh, Dhaka, Bangladesh. A copy of the English translation of the survey is available from the corresponding author on request.

### Measures

#### Sociodemographic factors

Sociodemographic information was collected regarding gender, educational status (e.g., university, medical college, high school), present residence (e.g., urban or rural), relationship status (i.e., single, in a relationship, married), monthly family income (e.g., lower-class = <15,000 BDT, middle class = 15,001–30,000 BDT, upper class = more than 30,000 BDT) and type of family (e.g., nuclear or extended family). Additionally, their current living condition with family was also assessed.

#### Behavioral health-related measures

Behavioral health related information was collected based on participants smoking status, sleep status, and physical exercise. For assessing sleeping patterns, the study followed prior Bangladeshi studies comprising three categories [e.g., normal sleeping status = 6–7 h (22)]. Physical exercise in the form of walking, cycling, swimming, or other activities for at least 30 min daily was considered. Perceived health status was assessed by asking participants whether they suffered from chronic diseases or not (e.g., asthma, diabetes, heart problems, kidney problems, etc.) (13, 23).

#### Online use behaviors

Several online use behaviors were assessed in the present study. Considering the prior Bangladeshi studies, the duration of online use was assessed utilizing categories (e.g., <2 h, 2–3 h, 4–5 h, and more than 5 h). The online activities included educational activities, chatting/texting, online gaming, watching/streaming videos/films, social media browsing, watching sexual materials/pornography, and online shopping (13, 23).

#### Depression

The two-item Patient Health Questionnaire (PHQ-2) was used for assessing the presence of depression. Participants are asked how often they experienced the two core criteria for depressive disorders over the past 2 weeks (i.e., “Little interest or pleasure in doing things”, and “Feeling down, depressed, or hopeless”), which are responded to on a 4-point Likert scale (0 = not at all, 1 = several days, 2 = more than half the days, 3 = nearly every day) (24, 25). The total score ranges from 0 to 6, where ≥ 3 was considered as the cutoff point indicating the presence of depression (25). In the present study, the Cronbach's alpha was 0.73.

#### Anxiety

The two-item Generalized Anxiety Disorder (GAD-2) scale was used for assessing the presence of anxiety. Participants are asked how often they experienced the two core criteria for anxiety disorders over the past 2 weeks (i.e., “Feeling nervous, anxious or on edge”, and “Not being able to stop or control worrying”), which are responded to on a 4-point Likert scale (0 = not at all, 1 = several days, 2 = more than half the days, 3 = nearly every day) (25, 26). The total score ranges from 0 to 6, where ≥ 3 was considered as the cutoff point indicating the presence of anxiety (25). In the present study, the Cronbach's alpha was 0.73.

#### Problematic Facebook use

The Bergen Facebook Addiction Scale was used for assessing problematic Facebook Use (27). The scale comprises six items (e.g., “How often in the last year have you spent a lot of time thinking about Facebook or planned use of Facebook?”), which are responded to on a 5-point Likert scale from 1 (very rarely) to 5 (very often). A score of ≥18 (out of 30) was used as cutoff score to operationally define problematic Facebook users (27) as has been used in previous studies in Bangladesh [e.g., (13, 21)]. In the present study, the Cronbach's alpha was 0.73.

### Statistical analysis

IBM SPSS Statistics for Windows (Version 25.0. Armonk, NY: IBM Corp.) and Microsoft Excel 2019 were used for statistical analysis. Descriptive statistics (such as frequency, and percentages, mean, and standard deviation), and inferential statistics were applied in the present study. The data distribution was normal and multi collinearity-related issues were absent. Therefore, parametric tests such as analysis of variance (ANOVA) test, and independent sample *t*-test were used considering the variables category to assess mean differences among the studied variables using Bonferroni correction, with *p* = 0.002 as the significance level. The Pearson correlation was used to establish the linear association between continuous variables and problematic Facebook use. A multivariable linear regression model was utilized to identify the predictive factors influencing problematic Facebook use with a 95% confidence interval.

## Results

### Characteristics of the participants

More than half of the participants were male (57.2%) and used the internet daily for more than 5 h (53.2%). Nearly two-thirds were university students (65.6%), more than one-quarter were medical college students (29.6%), and the remainder were high school students aged 18–19 years (4.8%). Approximately three-quarters came from an urban area (75.2%) and nuclear family (78%), and 44.6% had more than 30,000 (BDT) monthly income. Four-fifths of participants were single (79.5%). Approximately half of the participants (49.1%) took part in physical exercise and more than 90% used the internet for messaging, watching videos, and browsing social media. Additionally, 43.3% and 32.6% of the participants reported depression and anxiety symptomology, respectively (Table 1).

**Table 1:** Distribution of the studied variables with problematic Facebook use.

**Variables**	**Total sample**			
	***n*** **(%)**	**Mean** ±**SD (BFAS score)**	**F/** ***t*** **-test value**	***p*** **-value**
**Socio-demographic variables**				
**Gender**				
Male	344 (57.2)	15.86 ± 5.63	−0.719	0.473
Female	257 (42.8)	16.20 ± 5.82		
**Educational status**				
University	394 (65.6)	15.68 ± 5.41	10.923	<0.001
Medical college	178 (29.6)	17.29 ± 5.86		
High school	29 (4.8)	12.51 ± 6.80		
**Current residence**				
Rural	149 (24.8)	15.99 ± 5.43	−0.037	0.971
Urban	452 (75.2)	16.01 ± 5.81		
**Monthly family income (BDT)**				
<15,000	106 (17.6)	15.85 ± 5.64	1.212	0.298
15,000–3,000	227 (37.8)	16.46 ± 5.42		
>30,000	268 (44.6)	15.67 ± 5.97		
**Family type**				
Joint	132 (22.0)	15.99 ± 5.14	−0.036	0.971
Nuclear	469 (78.0)	16.01 ± 5.87		
**Relationship status**				
Single	478 (79.5)	16.19 ± 5.62	7.550	0.001
In a relationship	67 (11.1)	16.92 ± 5.63		
Married	56 (9.3)	13.30 ± 5.92		
**Currently living with family**				
No	78 (13.0)	15.56 ± 5.98	−0.735	0.462
Yes	523 (87.0)	16.07 ± 5.67		
**Behavioral health-related questions**				
**Number of daily sleeping hours**				
<6 h	69 (11.5)	15.47 ± 5.62	0.601	0.549
6–7 h	324 (53.9)	15.93 ± 5.48		
More than 7 h	208 (34.6)	16.30 ± 6.09		
**Physical exercise**				
No	306 (50.9)	16.95 ± 5.69	4.188	<0.001
Yes	295 (49.1)	15.02 ± 5.58		
**Smoking status**				
No	550 (91.5)	16.09 ± 5.62	1.163	0.245
Yes	51 (8.5)	15.11 ± 6.65		
**Perceived health related problem**				
No	536 (89.2)	15.92 ± 5.58	−0.865	0.390
Yes	65 (10.2)	16.67 ± 6.70		
**Online use behaviors**				
**Daily internet use time**				
<2 h	23 (3.8)	11.21 ± 3.94	21.561	<0.001
2–3 h	114 (19.0)	14.06 ± 5.06		
4–5 h	144 (24.0)	14.86 ± 4.87		
More than 5 h	320 (53.2)	17.55 ± 5.88		
**Purpose of online use (Yes vs. No)**				
Educational	506 (84.2)	15.89 ± 5.62 vs. 16.58 ± 6.17	1.080	0.281
Messaging	581 (96.7)	16.14 ± 5.61 vs. 12.15 ± 7.22	−2.444	0.024
Gaming	148 (24.6)	15.97 ± 5.80 vs. 16.01 ± 5.69	0.070	0.944
Watching video	556 (92.5)	16.09 ± 5.65 vs. 14.97 ± 6.42	−1.258	0.209
Social media use	574 (95.5)	16.29 ± 5.59 vs. 9.85 ± 4.80	−5.884	<0.001
Shopping	128 (21.3)	16.00 ± 5.82 vs. 16.01 ± 5.69	0.019	0.985
News	379 (63.1)	15.87 ± 5.61 vs. 16.23 ± 5.89	0.756	0.450
Others	405 (67.4)	16.20 ± 5.71 vs. 15.61 ± 5.90	−1.182	0.238
**Psychopathological factors**				
**Depression (Cutoff point:** **≥3 out of 6)**				
Probable depression	260 (43.3)	18.10 ± 5.67	−8.259	<0.001
Normal	341 (56.7)	14.41 ± 5.21		
**Anxiety (Cutoff point:** **≥3 out of 6)**				
Probable anxiety	196 (32.6)	19.09 ± 5.66	−9.588	<0.001
Normal	405 (67.4)	14.51 ± 5.11		

*The bold values referred to significant results*.

### Mean differences of studied variables with problematic Facebook use

The mean BFAS score was 16 out of 30 (SD ± 5.72) and slightly more than one-quarter (29.1%) were classified as problematic Facebook users (cutoff: ≥18). Table 1 showed no significant gender differences among the participants in terms of PFU. Medical students reported significantly higher PFU score than the other cohorts (*F* = 10.923, *p* < 0.001) whereas those in a relationship had significantly higher PFU score than single and married participants (*F* = 7.550, *p* = 0.001). PFU levels were significantly higher among participants who did not engage in physical exercise (*t* = 4.188, *p* < 0.001) and used the internet for more than 5 h daily (*F* = 21.561, *p* < 0.001). In relation to specific types of internet use, messaging (*p* = 0.024) and social media browsing (*p* < 0.001) were significantly associated with PFU. In addition, depression and anxiety symptomology were both significantly associated with PFU (*p* < 0.001).

### Correlation coefficient between continuous variables and problematic Facebook use

The result showed that PFU had a significant linear relationship with depression and anxiety. The relationship of PFU with depression (*r* = 0.411, *p* < 0.001) and anxiety (*r* = 0.460, *p* < 0.001) was moderately strong while depression and anxiety was strongly related (*r* = 0.630, *p* < 0.001) (Table 2).

**Table 2:** Correlation coefficients between continuous variables and problematic Facebook use.

**Variables**	**Mean ±SD**	**Problematic Facebook use**	**Depression**	**Anxiety**
Problematic Facebook use	16.0 ± 5.71	1		
Depression	2.37 ± 1.42	0.411^**^	1	
Anxiety	2.07 ± 1.57	0.460^**^	0.630^**^	1

** *Correlation is significant at the 0.01 level (2-tailed)*.

### Predictive models for problematic Facebook use

Table 3 presents predictive models for PFU using multivariable linear regression. The model predicted that increasing in daily internet use time (*B* = 1.293, *p* < 0.001), social media use (*B* = 5.297, *p* < 0.001), depression (*B* = 0.671, *p* < 0.001), and anxiety (*B* = 1.119, *p* < 0.001) can positively increase the PFU. Additionally, relationship status (*B* = −0.741, *p* = 0.020), and gaming (*B* = −0.982, *p* = 0.046) negatively impacted PFU. The overall model explained 33.2% variance for predicting PFU [F (18, 582) = 16.085, *p* < 0.001, *R*^2^ = 0.332].

**Table 3:** Predictive models for problematic Facebook use.

**Variables**	**Model Fit: F (18, 582)** = **16.085**, ***p**<* **0.001, R**^2^ = **0.332**				
	**B**	**Std. Error**	β	**95% CI (LB, UB) for B**	***p***
Constant	4.711	2.129		0.529, 8.893	0.027
Relationship status	−0.741	0.317	−0.082	−1.363, −0.118	0.020
Daily internet use time	1.293	0.228	0.203	0.845, 1.740	<0.001
Gaming	−0.982	0.492	−0.074	−1.948, −0.015	0.046
Social media use	5.297	1.021	0.192	3.291, 7.303	<0.001
Depression	0.671	0.179	0.167	0.319, 1.023	<0.001
Anxiety	1.119	0.162	0.308	0.801, 1.437	<0.001

*Only significant variables have been shown in the table. B = Unstandardized coefficient; β = Standardized coefficient; LB = Lower bound; UB = Upper bound*.

## Discussion

Using a cutoff score of ≥18 out of 30 on the Bergen Facebook Addiction Scale, 29.1% of the sample were operationally defined as problematic Facebook users. This prevalence rate of PFU is lower than the two previous Bangladeshi studies among Bangladeshi university students utilizing the same instrument [39.7% in (13), 36.9% in (21)]. No previous study has investigated PFU during the pandemic in Bangladesh. It was expected that students would be at higher risk of PFU given the higher exposure to the internet during the pandemic.

The lower prevalence of PFU in the present study may have been because students spent more time on other internet-related activities (such as online learning and teaching). Studies conducted outside of Bangladesh have also reported higher rate of problematic Facebook use among students than in the present study including Malaysia [47% of the university students (*n* = 441) (28)], and Thailand [41.8%; of the high school students (*n* = 972) (29)]. The prevalence rate of PFU in a study among postgraduate university students (*n* = 100) in India was 26% (30). The differences in prevalence rates may simply have been due to methodological factors such as the different samples, setting, and sample size.

Results showed no significant gender differences among the participants in respect to PFU. This concurs with the findings of the two previous Bangladeshi studies (13, 21). Relationship status was not significantly associated with PFU in previous Bangladeshi studies (13, 21), but a two-fold higher risk of PFU was found among students who had failed to initiate a romantic relationship (21). The present study found a significantly higher risk of PFU among participants currently in a relationship compared to those who were married or single. In-person interaction may have been restricted between such participants during the long-enforced lockdown. Therefore, they may have spent more time on Facebook maintaining communication with each other, which contributed to PFU.

The present study also found that medical students had a higher level of PFU than university or high school students which has not been explored in any previous studies. Facebook generally attracts students with its various features (e.g., communication, entertainment, and information exchange) particularly in the home-confined situation that occurred during the COVID-19 pandemic. In general, medical students have more stressful academic study than other students (31) which may result in students using Facebook primarily to relieve stressful academic pressures. Furthermore, in order to connect with friends or family members, Facebook use may result in students spending increasingly more time on the site, leading to problematic or addictive behavior for some. The present study also suggested that high school students reported less PFU. This may be because they are restricted in using their smartphones by their parents in Bangladeshi culture.

Predictably, and as with previous Bangladeshi studies (13, 21) spending more time on internet was associated with PFU in the present study. Given that individuals are spending more time on Facebook, it is likely that they are using the platform for various online purposes (e.g., messaging friends, watching videos, reading news, etc.,). The online use of messaging and social media browsing was predictably associated with problematic Facebook use given that Facebook use comprises both these online activities, but educational use of the internet was not associated with PFU in the present study. It is evident that PFU can adversely affect a user's mental health and has been regarded as a global public health concern among a minority of social media users (18, 20). As expected, depression and anxiety symptomology had the strongest associations with PFU. Previous studies have indicated that PFU is associated with depression (32–34). Prior Bangladeshi studies have reported that depressed students are approximately at two to three times greater risk of PFU (13, 21). Similarly, other studies have reported anxiety as a predictor of PFU (35–37). Studies have also shown that trait anxiety is a positive predictor for PFU in the US (36), Pakistan (37), and other countries (18).

The present study findings also concur with previous research that depression and anxiety (as assessed using the PHQ-2 and GAD-2 scales) were significantly associated with PFU during COVID-19 pandemic, indicating that students with depression and anxiety who uses Facebook are at increased risk of developing PFU. However, the study did not directly ask students if their depression and anxiety symptoms had worsened during the pandemic. As a result, future research should determine whether an increase in PFU is associated with an increase in reported depression or anxiety symptoms.

The present study has some limitations. As the study was conducted online, used convenience sampling, and had a modestly sized sample, various selection biases may have arisen. Moreover, the study was non-representative in nature as only participants from a few higher educational establishments were recruited, some types of students were not represented (e.g., there were no engineering students and specific study disciplines were not asked in the survey), all participants were recruited online, and the data were self-report, all of which have well-known methods biases. Finally, it should also be noted that although the BFAS has been used in Bangladesh in a few previous studies it has not been officially validated into Bangla.

## Conclusion

The study found that 29.1% students were problematic Facebook users. Medical college students had higher score on PFU than others, whereas relationship status, daily internet use time, social media use, and psychological symptoms were the predictive factors for PFU. Although this study reported lower prevalence rate of PFU than the prior Bangladeshi studies, the proportion of students at risk of PFU was still arguably of concern. Educational institutes should implement interventions to reduce PFU among students. Additionally, therapeutic interventions can be developed for healthy and safe Facebook use with a focus on student mental health given its association with PFU.

## Data availability statement

The raw data supporting the conclusions of this article will be made available by the corresponding author upon reasonable request.

## Ethics statement

The studies involving human participants were reviewed and approved by Institute of Allergy and Clinical Immunology of Bangladesh, Dhaka, Bangladesh (Reference: IRBIACIB/CEC/03202030). The patients/participants provided their written informed consent to participate in this study.

## Author contributions

FA-M and MM conceptualized the study and wrote the first draft, whereas all author contributed to revise the manuscript. FA-M, IH, and MM partook in study implementation, data collection, and data analysis. MG and MM supervised the project. All authors contributed to the article and approved the submitted version.

## Conflict of interest

The authors declare that the research was conducted in the absence of any commercial or financial relationships that could be construed as a potential conflict of interest.

## Publisher's note

All claims expressed in this article are solely those of the authors and do not necessarily represent those of their affiliated organizations, or those of the publisher, the editors and the reviewers. Any product that may be evaluated in this article, or claim that may be made by its manufacturer, is not guaranteed or endorsed by the publisher.
